# The detection of faked identity using unexpected questions and choice reaction times

**DOI:** 10.1007/s00426-020-01410-4

**Published:** 2020-09-04

**Authors:** Merylin Monaro, Ilaria Zampieri, Giuseppe Sartori, Pietro Pietrini, Graziella Orrù

**Affiliations:** 1 Department of General Psychology, University of Padua, Padua, Italy; 2 IMT School for Advanced Studies, Lucca, Italy; 3 Department of Surgical, Medical and Molecular Pathology and Critical Care Medicine, University of Pisa, Pisa, Italy

## Abstract

The identification of faked identities, especially within the Internet environment, still remains a challenging issue both for companies and researchers. Recently, however, latency-based lie detection techniques have been developed to evaluate whether the respondent is the real owner of a certain identity. Among the paradigms applied to this purpose, the technique of asking unexpected questions has proved to be useful to differentiate liars from truth-tellers. The aim of the present study was to assess whether a choice reaction times (RT) paradigm, combined with the unexpected question technique, could efficiently detect identity liars. Results demonstrate that the most informative feature in distinguishing liars from truth-tellers is the Inverse Efficiency Score (IES, an index that combines speed and accuracy) to unexpected questions. Moreover, to focus on the predictive power of the technique, machine-learning models were trained and tested, obtaining an out-of-sample classification accuracy of 90%. Overall, these findings indicate that it is possible to detect liars declaring faked identities by asking unexpected questions and measuring RTs and errors, with an accuracy comparable to that of well-established latency-based techniques, such as mouse and keystroke dynamics recording.

**Electronic supplementary material:**

The online version of this article (10.1007/s00426-020-01410-4) contains supplementary material, which is available to authorized users.

## Introduction

Millions of people have their identities stolen every year. There is no fool-proof way to pinpoint fakers, especially when faked identities are used to register online. Traditional methods of lie detection include face-to-face interviews and polygraphs that measure heart rate and skin conductance (Granhag, Vrij, & Verschuere, 2015). Leaving aside the debated accuracy of the polygraph, these techniques cannot be used remotely or with large numbers of people.

Recently, researchers have developed latency-based measures to determine whether the respondent is the real owner of a certain identity (Sartori, Zangrossi, & Monaro, 2018). Latency-based lie detection techniques find their roots in the cognitive load theory, according to which lying requires a greater cognitive effort than truth-telling; this higher workload is reflected by a number of indices, including, for example, reaction times (RT) (Vrij, Fisher, & Blank, 2015). Indeed, people show an increase in RT and error rate when they lie in response to questions (Vrij, Fisher, Mann, & Leal, 2008). So far, RT-based techniques have been almost exclusively tested in the laboratory setting, without taking into account some variables that could limit the application in a real scenario (e.g., the presence of interfering stimuli that can distract the participant and affect the response time). Although these techniques are still far from finding an application in ecological contexts, different proofs of concept demonstrated the feasibility of this approach for false identity detection.

Verschuere and Kleinberg used the CIT-RT technique to evaluate whether respondents were lying about their identity (Verschuere & Kleinberg, 2016). The CIT-RT consists in presenting critical information (the concealed information which is known only by the guilty subject) within a series of similar but noncritical information (stimuli which are irrelevant both for guilty and innocent subjects). The aim of this technique is to evaluate if the examinee recognises specific information through indirect measures (Verschuere, Ben-Shakhar, & Meijer, 2011). When applied to verify the autobiographical information that the examinee claims to correspond to the true identity, CIT efficiently succeeds in distinguishing the identities of liars and truth-tellers.

Monaro et al. compared performance of mouse-guided responses to true and false questions about identity (Monaro, Gamberini, & Sartori, 2017a, c; Monaro, Fugazza, Gamberini, & Sartori, 2017b). They asked liars to study a new identity (name, surname, date of birth, place of birth, place of residence) and to respond pretending that these faked identities were their true ones. Findings revealed that lying triggered a more erratic mouse trajectory and longer RTs, especially when questions about identity were unexpected. Indeed, whereas unexpected questions about identity, such as the Zodiac, can be easily addressed by a truth-teller, they typically require a deceptive subject to engage in mental computations to come up with the correct information (e.g., the correct zodiac corresponding to the false date of birth). The authors capitalized on unexpected questions that do not permit liars to prepare themselves and to anticipate a response to a predictable question (Hartwig, Granhag, & Strçmwall, 2007). Indeed, planning makes lying easier and planned lies typically contain fewer cues to deceit than do spontaneous lies (DePaulo et al., 2003). In line with this, the analyses based on expected questions correctly discriminated liars from truth-tellers with accuracies ranging from 65 to 67%, whereas the classification based on unexpected questions reached a 95% accuracy (Monaro et al., 2017b).

The efficiency of unexpected questions in detecting faked identities was proved in two additional studies in which the authors applied this technique to analyse the keystroke dynamics while participants were engaged in typing their personal information on the computer keyboard (Monaro et al., 2018, 2019). Like for the mouse dynamics, liars took more time to type their responses, especially to unexpected questions.

Thus, based on the fact that changes in mouse trajectory and keyboard dynamics as well as changes in response time were relevant in distinguishing liars from truth-tellers (Monaro et al., 2017b), here we investigated whether combining a choice reaction time paradigm with the technique of unexpected questions could efficiently detect individuals lying about their identity.

## Materials and methods

### Participants

Fifty native Italian-speaking individuals (23 males and 27 females) took part in the experiment. Power calculations indicated that a sample size = 50 in a between-subject design (*n* = 25 for each group) would have been sufficiently large to achieve at least a statistical power (1 − *β*) = 0.90, given a significance level (*α*) = 0.05 and an effect size (Cohen’s *d*) = 2.33 (note that the effect size is referred to the variable IES unexpected) (Faul et al., 2007). Most participants were students and were recruited at the University of Padua (Italy). They were all volunteers over 18 years of age. All of them provided a written informed consent before the experiment and did not receive any monetary compensation for the participation. Inclusion criteria were age equal or greater than 18 years and being native Italian speakers, to exclude any influence in response times due to reading or comprehension difficulties, as the experiment was run in the Italian language.

Data collected from 40 participants were used as a training set to build machine learning (ML) models and data from the remaining 10 subjects were used as a test set, to evaluate the model generalization capabilities. The demographic data of training and test samples are reported in Table 1.

**Table 1:** Demographic information about training and test set

Sample	Group	*N*	Gender	Age	Education
Training set		40	M = 19, F = 21	M = 22.3, SD = 1.4	M = 16.3, SD = 1.1
	Liars	20	M = 9, F = 11	M = 22.3, SD = 1.6	M = 16.2, SD = 1.1
	Truth-tellers	20	M = 10, F = 10	M = 22.3, SD = 1.3	M = 16.4, SD = 1.1
Test set		10	M = 4, F = 6	M = 24.4, SD = 3.3	M = 16.6, SD = 1.6
	Liars	5	M = 3, F = 2	M = 23.4, SD = 1.8	M = 16.4, SD = 1.1
	Truth-tellers	5	M = 1, F = 4	M = 25.4, SD = 4.4	M = 16.8, SD = 2.2

The second column (*N*) reports the number of participants for each sample. The third column shows the number of males and females in the training and test sets, respectively. The fourth and the fifth column report mean (M) and standard deviation (SD) for age and education

Participants were randomly assigned either to the liar or to the truth-teller group. Therefore, half of the sample performed the task as liars and the other half performed the task as truth-tellers.

### Experimental procedure

The Ethics Committee for Psychological Research at the University of Padua approved the experimental procedure.

The experimental procedure was similar to that previously reported (Monaro et al., 2017b). Participants assigned to the experimental condition (liar group), were asked to learn a fake identity profile that included faked first name, family name, date and place of birth, residence address, profession and civil state. Participants were required to rehearse the fake information until they were able to recall them by heart with no mistakes. Between the rehearsals, they were required to solve mathematical and logic tasks, to increase the cognitive load and to distract them from the learned information. This procedure was adopted to make sure that the fake identity profile was not stored merely in the working memory, but also in the long-term memory so that subjects could recall their fake identity for the whole duration of the experiment. Two distinct experimenters conducted the two phases of the experimental procedure. The first one assisted the subjects in memorizing their fake identity profile, as described above, while the second experimenter was in a separate room and gave instructions on how to perform the computerized task. The peculiarity of this procedure is that a “fake-blind” condition was created. The first experimenter (the one who trained the subjects) told the participants that the other researcher was not aware of the condition of each subject (liar or truth-teller). In this way, participants of the experimental condition were invited to do their best to cheat the second experimenter.

Participants assigned to the truth-teller group were asked to fill in their own data in a facsimile of an Italian identity card (ID). They were required to solve the same mathematical and logic tasks as the liar group, to balance the cognitive load before undertaking the computerized task. They repeated their personal data (name, surname, date and place of birth, residence address, profession and civil state) only once after the distracting mathematical riddles.

After the learning phase, both liars and truth-tellers entered the room where the second experimenter was allocated. They were required to show their fac-simile IDs and to wait a few minutes while the second experimenter entered their personal data (real or fake) into the system. Finally, they were asked to complete the computerized task that required them to respond to questions about their identity. The experiment was programmed in E-Prime^®^ 2.0 (Schneider, Eschman, & Zuccolotto, 2007). The experiment was run on a single laptop ASUS K56C with a 15.6″ diagonal screen LCD.

### Stimuli

Each participant was required to respond to 78 questions in total, including 18 control questions, 20 expected questions and 40 unexpected questions. All the questions were in the form of affirmative sentences that required a "yes" or "no" response, with a perfect balance, so that half of them required a “yes” response and the other half a “no” response, for both liars and truth-tellers.

The expected questions consisted of personal information provided by each subject in their ID, such as first name, family name, date and place of birth, residence address, profession and civil state. Liars expected to be tested on these pieces of information, as the first experimenter had made sure that each liar could perfectly recall the new identity and recommended each of them to cheat the second experimenter.

The unexpected questions concerned information that, though not explicitly presented in the IDs, could be extracted from the basic information contained in the IDs. Truth tellers did not need to think about the right answers to the unexpected questions, as extracting derived-from-the-ID-data information was an automatic and easy process for them. For instance, if you were born on April 20th, you should also know that your zodiac is Aries; if you lived in Padua, you should know the area zip code. By contrast, as the liars had learned their fake ID data, they needed a much greater cognitive effort and a longer time to answer unexpected questions. As a result, the liars showed increased response times and higher errors rates.

Finally, the control questions included some objective features of the participants that were directly verifiable by the experimenter, such as their gender, hair and eye colour or what they were wearing during the experiment.

In summary, each subject responded to 9 control questions requiring a “no” response, 9 control questions requiring a “yes” response, 10 expected questions requiring a “no” response, 10 expected questions requiring a “yes” response, 20 unexpected questions requiring a “no” response and 20 unexpected questions requiring a “yes” response. Examples of questions are reported in Table 2.

**Table 2:** The table reports examples of the 78 expected, unexpected and control questions presented to the participants and related to a true or fake identity

Type of question	Question that requires “yes” response by both liars and truth-tellers	Question that requires “no” response by both liars and truth-tellers
Expected	My name is Alice	My name is Maria
	My last name is Rossi	My last name is Bianchi
	I was born in 1989	I was born in 1986
	I was born in April	I was born in August
	I was born on 20th	I was born on 13th
	I was born in Mestre	I was born in Capri
	I live in Limena	I live in Caserta
	I live at Vespucci street	I live at Marconi street
	I am single	I am married
	I am a student	I am a professor
Unexpected	I am 27 years old	I am 23 years old
	My zodiac is Aries	My zodiac is Leo
	I was born in Veneto	I was born in Campania
	I was born in the province of Venice	I was born in the province of Napoli
	I live in Veneto	I live in Campania
	I live in the province of Padova	I live in the province of Caserta
	Venezia is the capital of the region where I live	Napoli is the capital of the region where I live
	Venezia is the capital of the region where I was born	Napoli is the capital of the region where I was born
	My first name contains double letters	My first name is without double letters
	The initials of my name are A.R	The initials of my name are M.B
	I already celebrated the birthday this year	I have yet to celebrate the birthday this year
	My last name contains double letters	My last name is without double letters
	My age minus one year is 26	My age minus one year is 25
	The city where I was born is just north of Bologna	The city where I was born is just south of Roma
	My zip code is 35142	My zip code is 7863
	My telephone area code is 049	My telephone area code is 062
	I live near the sea	I live near the mountains
	I live in the same region where I was born	I live in a different region than where I was born
Control	I live between Treviso and Rovigo	I live between Lucca and Arezzo
	I was born near Venice	I was born near Torino
	I am female	I am male
	My skin is white	My skin is brown
	I have a ring on my finger	My fingers are without rings
	I have light eyes	I have dark eyes
	I wear glasses	I am without glasses
	I am wearing a green t-shirt	I am wearing a blu t-shirt
	I am 160 cm high	I am 190 cm high
	I am attending the university	I am attending the high school
	I am wearing pants	I am wearing a skirt

Stimuli appeared in random order in the center of the computer screen and two response labels were placed, respectively, in the right and in the left upper corners of the screen. To give their response, subjects were instructed to press either the key “A” or the “L” on the computer keyboard that corresponded respectively to the left and the right response label. Moreover, they were instructed to press the response key on the computer keyboard as fast as they could and, at the same time, they had to try to be as much accurate as they could. Each stimulus appeared automatically after the response to the previous one, so no action was required to the subjects to bring up each new question. No temporal response limit was fixed and no feedback was provided for responses.

### Latency-based measures

During the task, we recorded RTs and numbers of errors. For errors, we mean the wrong responses provided by subjects according to the information that they reported, independently from the fact that they were liars or truth-tellers. Then, for each participant we computed the average RTs and the average number of errors, separately for control, expected and unexpected questions. Moreover, RTs were calculated separately for wrong and right responses. Then, we calculated the Inverse Efficiency Score (IES), an index that combines speed and accuracy (Bruyer & Brysbaert, 2011). As a matter of fact, subjects can increase the response speed during the task, but this usually leads to a higher proportion of error (PE). The IES considers the number of errors and increases proportionally the average RT of the subject according to the following formula:

$$\mathrm{IES}=\frac{\mathrm{RT}}{(1-\mathrm{PE})}$$

Equation 1: Calculation of the Inverse Efficiency Score.

The IES was calculated separately for control, expected and unexpected questions.

The final list of predictors is the following: RT control, RT expected, RT unexpected, RT control right responses, RT expected right responses, RT unexpected right responses, RT control wrong responses, RT expected wrong responses, RT unexpected wrong responses, errors control, errors expected, errors unexpected, IES control, IES expected, IES unexpected.

## Analyses and results

### Feature selection

Feature selection consists in the process of automatically selecting the best subset of predictors, to maximize the model accuracy, that is, in our specific case, the accuracy in discriminating between liars and truth-tellers. Feature selection is a widely used procedure in machine learning (ML) (Hall, 1999), as it allows to remove redundant and irrelevant features and to increase the model generalization by reducing over-fitting and noise in the data (Bermingham et al., 2015). Here, feature selection was performed using a correlation-based feature selector (CFS) algorithm, as implemented in WEKA 3.9 (Hall et al., 2009) and was applied to the original set of predictors (RT control, RT expected, RT unexpected, RT control right responses, RT expected wrong responses, RT unexpected right responses, RT control wrong responses, RT expected wrong responses, RT unexpected wrong responses, errors control, errors expected, error unexpected, IES control, IES expected, IES unexpected) using a tenfold cross-validation procedure. The CFS algorithm identifies the best subset of features by considering the individual predictive ability of each predictor, along with the degree of redundancy with the other predictors. The subsets of features that are highly correlated with the class (the dependent variable; in our case truth-tellers vs liars) and, at the same time, lesser inter-correlated with each other, are preferred. To search the subset of predictors through the spaces of features, the Greedy Stepwise search method was chosen (with forward search). We finally retained the four features most frequently selected in the tenfold: RT wrong expected (*r*_pb_ = 0.51, selected in four out of tenfold of the cross-validation), RT wrong unexpected (*r*_pb_ = 0.19, selected in ten out of tenfold of the cross-validation), IES expected (*r*_pb_ = 0.54, selected in nine out of tenfold of the cross-validation), IES unexpected (*r*_pb_ = 0.77, selected in ten out of tenfold of the cross-validation). Note that responses to control questions were discarded by the feature selection algorithm, as they did not carry any useful information to distinguish the two groups (truth-tellers vs liars).

Table 3 reports the correlation matrix of the four selected features and their correlation with the dependent variable (liar vs truth-teller).

**Table 3:**
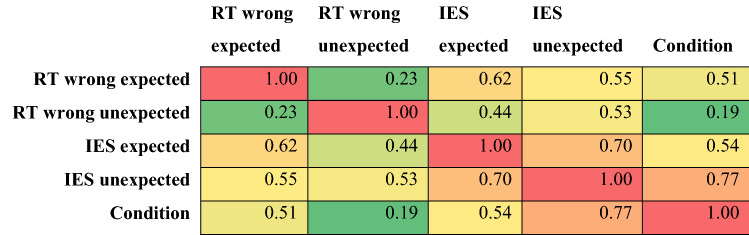
The table reports the correlation matrix for the four selected features and their correlation value with the dependent variable

### Descriptive statistics and analysis of variance

Table 4 reports the descriptive statistics for the four selected features in the original sample of 40 participants. It is worth to notice that liars provided on average 0.95 (SD = 0.89) wrong responses to expected questions and 12.95 (SD = 3.94) wrong responses to unexpected questions, while truth-tellers gave on average just 0.15 (SD = 0.37) wrong responses to expected questions and 4.45 (SD = 2.82) wrong responses to unexpected questions.

**Table 4:** Descriptive statistics for the features selected by the CFS algorithm (RT wrong expected, RT wrong unexpected, IES expected, IES unexpected)

Feature	Group	M (SD)	Cohen’s *d*
RT wrong expected	Liars	1236.62 (1134.05)	1.17
	Truth-tellers	195.9 (481.10)	
RT wrong unexpected	Liars	3134.65 (1103.06)	0.39
	Truth-tellers	2613.94 (1609.95)	
IES expected	Liars	1896.42 (392.57)	1.26
	Truth-tellers	1451.06 (288.54)	
IES unexpected	Liars	4463.56 (1325.12)	2.33
	Truth-tellers	2195.06 (332.37)	

For each feature, the Cohen’s *d* value differentiating liars from truth-tellers is also reported

An ANOVA was run to investigate the difference between the two experimental groups (liars vs. truth-tellers), both for RT in wrong responses and IES. RTs to wrong responses of liars were longer than those of truth tellers [*F*_(1,38)_ = 7.80, *p* < 0.01, *η*^2^ = 0.06]. In addition, both liars and truth-tellers had longer RT in responding to unexpected questions compared to expected questions [*F*_(1,38)_ = 77.31, *p* < 0.01, *η*^2^ = 0.44]. No statistically significant results emerged from the interaction condition (liars vs. truth-tellers) X type of question (expected vs. unexpected).

As far as IES is concerned, ANOVA indicated that liars had a greater IES than truth-tellers [*F*_(1,38)_ = 51.06, *p* < 0.01, *η*^2^ = 0.25]. Moreover, both liars and truth-tellers had greater IES in responses to unexpected questions compared to expected questions [*F*_(1,38)_ = 151.60, *p* < 0.01, *η*^2^ = 0.36]. Finally, the interaction condition (liars vs. truth-tellers) X type of question (expected vs. unexpected) was statistically significant [*F*_(1,38)_ = 47.04, *p* < 0.01, *η*^2^ = 0.11]. Indeed, data showed a larger difference between liars and truth-tellers based on unexpected compared to expected questions (see Cohen’s *d* values in Table 4).

Analyses were run using “ez” package in R software (2016).

### Machine learning models

In the last years, researchers from different scientific fields have emphasized the utility of focus on prediction rather than explanation when data are analysed (Yarkoni & Westfall, 2017). Attention to predictive models has increased mainly thanks to the significant spread of machine learning (ML) techniques, which allow to train algorithms on samples of data (training set) to make predictions on completely new data (test set) without being explicitly programmed (Orrù et al., 2020). As far as psychology is concerned, ML techniques are particularly useful to predict human behaviour, including deception (Zago, Piacquadio, & Monaro, 2019). Indeed, ML makes it possible to draw inferences at the individual level, while traditional statistical methods focus on a group level. Thus, by applying ML models, one can assess individual subject behaviour.

**Fig. 1:**
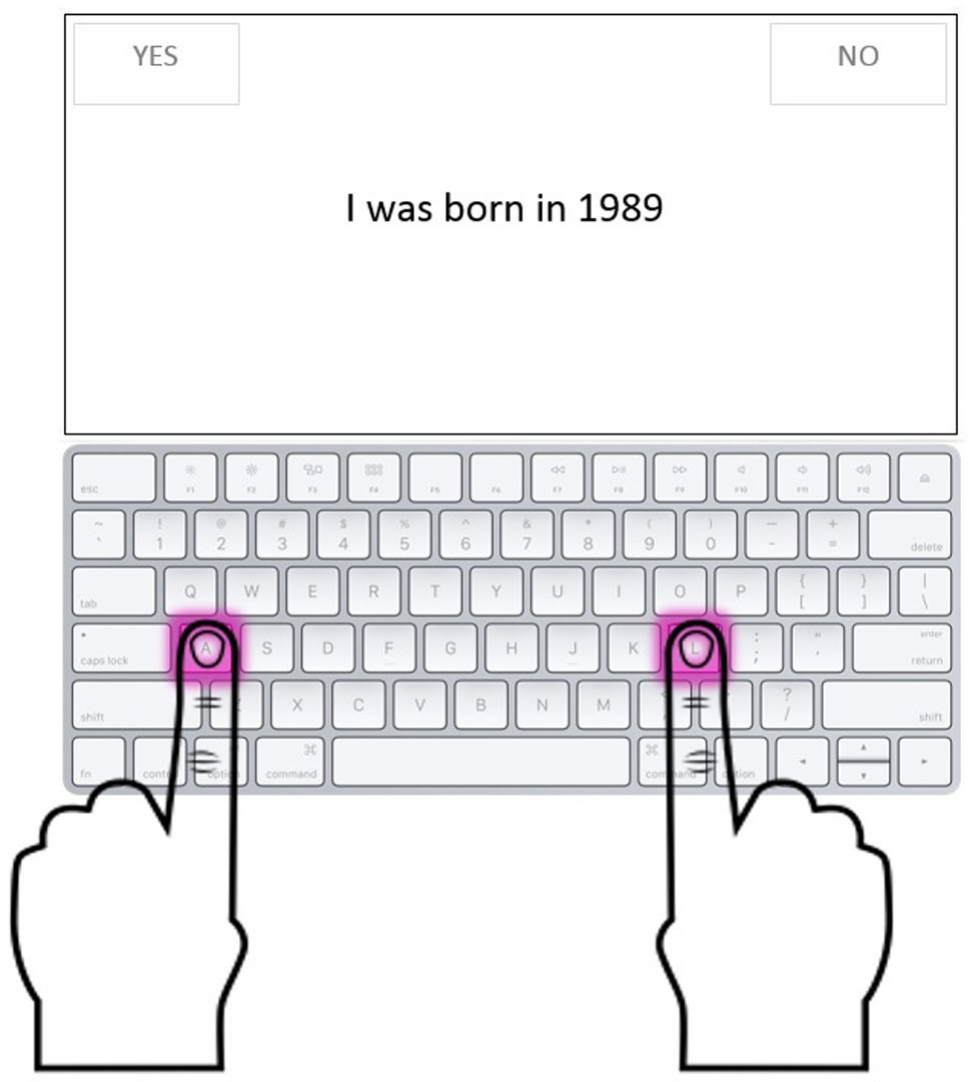
The figure shows an example of the computerized experimental task

The four selected features (RT wrong expected, RT wrong unexpected, IES expected, IES unexpected) were entered in five different ML algorithms: Logistic (le Cessie & van Houwelingen, 1992), SVM (Keerthi, Shevade, Bhattacharyya, & Murthy, 2001), Naïve Bayes (John & Langley, 1995), Random Forest (Breiman, 2001), LMT (Landwehr, Hall, & Frank, 2005). A useful strategy to avoid cherry peaking the best performing model is to verify that classification accuracy does not change significantly among different classes of classifiers (Orrù et al., 2020). If similar results are obtained by ML models relying on radically different assumptions, one may be relatively confident that results are not dependent on specific assumptions. For this reason, we developed the five models mentioned above. (Fig. 1)

All models were validated following a tenfold cross-validation procedure (Kohavi, 1995). Cross-validation is a resampling procedure used to reduce variance in the model performance estimation. The procedure uses parameter *k*, where *k* is a positive integer and splits the data set into *k* groups. One group is used as a hold out of the validation set, while the rest is used to train the model. Next, we trained our model on the training set and evaluated the performance of the validation set. We kept the score of each validation, reshuffled the data set randomly and repeated the procedure for *k* times, hence the name *k*-fold cross validation.

Finally, the five models, which were validated through the tenfold cross-validation procedure, were tested on a new sample of 10 participants. Indeed, as ML models are built to fit the data, it is important to test how an existing model fits new unseen data. For this reason, part of the data (training set, *n* = 40) was used to train and validate the model, while another part (test set, *n* = 10) was set aside to test the model accuracy on new examples that had never been seen by the ML classifier (Nelles, 2001). This procedure guarantees the generalization of the model and increases the replicability of results (Cumming, 2008; Dwork et al., 2015), a crucial issue in behavioral experiments (Baker, 2016).

Results obtained by the tenfold cross-validation procedure are reported in Table 5.

**Table 5:** The table reports the accuracy obtained by five classifiers in correctly identifying liars and truth-tellers, in the training and test sets

ML classifier	Training set (tenfold cross-validation)			Test set		
	Average accuracy (SD)	FP rate (%)	FN rate (%)	Accuracy (%)	FP rate (%)	FN rate (%)
Logistic	90% (12.9)	10	10	80	20	20
SVM	90% (17.7)	0	20	90	0	20
Naïve Bayes	90% (17.5)	20	0	90	20	0
Random forest	97.5% (7.9)	0	5	90	20	0
LMT	95% (10.5)	0	10	90	20	0

The accuracy in the training set, using a tenfold cross-validation, is the average accuracy resulting from the tenfold. The standard deviation (SD) of the tenfold is also reported. False positive (FP) are the number of truth-tellers misclassified as liars, while false negative (FN) are the number of liars misclassified as truth-tellers

Finally, we tested the generalization of the model performance on the new set of ten participants who had not been included in the development of ML models. The results confirmed that all the models reached an accuracy of about 90% in classifying subjects as liars or as truth-tellers, both in training and test (see Table 5). The comparable results between the tenfold cross-validation and the test set indicated that cross-validation is a valid conservative estimate of the replicability power of the model. Moreover, the relatively constant performance on the out-of sample 10 participant test set indicated that the estimate accuracy does not depend on the specific assumptions of the models.

About the rate of false positive and false negative, the confusion matrix showed that the number of misclassified liars and truth-tellers was not equal for all the algorithms. Logistic regression produced a balanced number of false positives and false negatives, failing in detecting two liars and two truth-tellers in the training set and one liar and one truth-teller in the test set. The SVM was completely unbalanced towards the false negatives, misclassifying four liars in the training set and one liar in the test set. Naïve Bayes had an opposite performance, failing the classification of four truth-tellers in the cross-validation and one truth-teller in the test set. Random forest misclassified only one liar in the training set and one truth-teller in the test set. Finally, logistic model tree (LMT) failed in recognizing two liars in the cross-validation procedure and one truth-teller in the test set.

## Discussion

Detecting liars of personal identities is becoming an increasing important goal, as faked identities plague more and more the web and social networks. This is due to the fact that personal identities information can be easily learned and rehearsed to a point that lies are expressed as naturally and automatically as truths. Different researches capitalised on the use of unexpected questions and the use of mouse or keystroke dynamics to overcome automaticity in rehearsed lies (Monaro et al., 2017b). The present experiment expanded previous research using choice reaction times in reply to statement like questions requiring “yes” and “no” responses.

We developed ML classifiers to evaluate the out-of-sample accuracy of the models in distinguishing liars from truth-tellers. Machine learning was used to complement the standard statistical analyses for the following reasons (Orrù et al. 2020):

- ML models focus on the predictive power of the models and most lie detection research is about accuracy in spotting liars.
- ML models allow to estimate out-of-sample accuracy.

The results reported here confirm that the most informative features in distinguishing between liars and truth-tellers are the IES and the RT to wrong responses, both to expected and unexpected questions. More precisely, our analysis indicated that:

- RT-based test of liars about identity had a similar accuracy as mouse-based or keystroke dynamics-based detection (all these techniques reached at least 90% of accuracy in the test set).
- The most relevant predictor that contributed to detecting liars was IES (a measure that is intended to handle speed-accuracy trade-off). Moreover, as concerns IES, the differentiation between liars and truth-tellers is much stronger with unexpected compared to expected questions, confirming that using unexpecting questions is a promising approach.
- The time taken to wrongly respond to expected and unexpected questions also contributed to the classification model performance. It should be noticed that truth-tellers had very short RTs when they gave wrong responses to expected questions. This indicates that when a truth-teller fails in responding to expected questions, this is probably due to the speeded impulsivity in the response. On the other hand, the errors of the liars likely were due to incapacity to retrieve the correct information.
- The results are not model-dependent, as a variety of ML models that rely on very different assumptions performed at similar levels of accuracy.

To conclude, it is possible to spot liars declaring faked identities by asking unexpected questions and measuring RT and errors with an accuracy equivalent to that of mouse and keystroke dynamics recording (Monaro et al., 2017b, 2018). While the overall accuracy achieved with choice reaction times and mouse dynamics is comparable, mouse dynamics seems more resistant to countermeasures, as many parameters must be kept under control at the same time to fake the results (Monaro et al., 2017b). Countermeasures are strategies implemented by the liars to avoid detection. As detection of liars using mouse dynamics is based on a multitude of parameters that encode timing and erraticism of the mouse movement, they are more likely to be resistant to explicit strategies to doctor the results.

On the other hand, the advantages of using choice reaction time, as reported here, are that the experiment is simpler to build and to analyse. In addition, in this case countermeasures are not easy to develop without an explicit coaching aimed at selectively teach the cheater to modify the latencies in responding to erroneous responses.

As it is the case for a mental chronometric approach to lie detection in general, the current main limitation is represented by the difficulty to apply this technique in ecological contexts, in which the subject behaviour is not under the researcher control. What is different from the laboratory to the daily reality is the number of external stimuli, which may interfere with the task and which can lead to wrong conclusions. Indeed, any other activity that may be usually carried out by individuals during an ID recording, as well as problems with bandwidth, could result in longer response times and, therefore, may produce false-positive liars. Therefore, to further evaluate this approach, future experiments should be conducted by recruiting participants via the Web in a more ecological setting. A first attempt in this direction has already been done by Monaro et al. who measured temporal keystroke features when participants were asked to fill an online form with their real or faked identity information (Monaro et al., 2018). The classification model built on a first sample of participants who were recruited in the laboratory showed a high generalization to a second sample of participants who were recruited via the Web, reaching high accuracy also in the ecological setting (89–94%).

## Availability of data and material

Data are available as supplementary material.

## Electronic supplementary material

Below is the link to the electronic supplementary material.

Supplementary file1 (XLSX 12 kb)

## Data Availability

None.
